# Enlarging a training set for genomic selection by imputation of un-genotyped animals in populations of varying genetic architecture

**DOI:** 10.1186/1297-9686-45-12

**Published:** 2013-04-26

**Authors:** Eduardo CG Pimentel, Monika Wensch-Dorendorf, Sven König, Hermann H Swalve

**Affiliations:** 1 Department of Animal Breeding, University of Kassel, 37213, Witzenhausen, Germany; 2 Institute of Agricultural and Nutritional Sciences, University of Halle, 06099, Halle, Germany

## Abstract

**Background:**

The most common application of imputation is to infer genotypes of a high-density panel of markers on animals that are genotyped for a low-density panel. However, the increase in accuracy of genomic predictions resulting from an increase in the number of markers tends to reach a plateau beyond a certain density. Another application of imputation is to increase the size of the training set with un-genotyped animals. This strategy can be particularly successful when a set of closely related individuals are genotyped.

**Methods:**

Imputation on completely un-genotyped dams was performed using known genotypes from the sire of each dam, one offspring and the offspring’s sire. Two methods were applied based on either allele or haplotype frequencies to infer genotypes at ambiguous loci. Results of these methods and of two available software packages were compared. Quality of imputation under different population structures was assessed. The impact of using imputed dams to enlarge training sets on the accuracy of genomic predictions was evaluated for different populations, heritabilities and sizes of training sets.

**Results:**

Imputation accuracy ranged from 0.52 to 0.93 depending on the population structure and the method used. The method that used allele frequencies performed better than the method based on haplotype frequencies. Accuracy of imputation was higher for populations with higher levels of linkage disequilibrium and with larger proportions of markers with more extreme allele frequencies. Inclusion of imputed dams in the training set increased the accuracy of genomic predictions. Gains in accuracy ranged from close to zero to 37.14%, depending on the simulated scenario. Generally, the larger the accuracy already obtained with the genotyped training set, the lower the increase in accuracy achieved by adding imputed dams.

**Conclusions:**

Whenever a reference population resembling the family configuration considered here is available, imputation can be used to achieve an extra increase in accuracy of genomic predictions by enlarging the training set with completely un-genotyped dams. This strategy was shown to be particularly useful for populations with lower levels of linkage disequilibrium, for genomic selection on traits with low heritability, and for species or breeds for which the size of the reference population is limited.

## Background

Prediction of breeding values of animals using genomic information was proposed by Meuwissen et al. [1] and since then the way breeding programs of livestock are conducted has changed considerably. Due to recent advances in genotyping technologies, the amount of genomic information available for genomic selection (GS) has increased from a few thousand [2] to 50k [3] and 800k [4] single nucleotide polymorphism (SNP) markers and today tends towards whole-genome sequence [5]. The population structures observed in many livestock species are often characterized by large full- and half-sib families, and by the presence of animals (especially males) with a very large number of progeny. These conditions make it possible to infer the genotype of an un-genotyped individual using genomic information from its family members, which is usually referred to as pedigree-based imputation. High levels and extents of linkage disequilibrium (LD) have been reported in livestock populations, such as cattle [6], sheep [7], chickens [8], pigs [9] and horses [10]. The presence of high LD between markers can be used to infer the genotype at an un-genotyped locus based on available genotypes at neighbouring markers, which is usually referred to as population-based imputation. Such features make it possible to impute genotypes at untyped markers in a larger panel of markers from genotypes obtained with a smaller panel. In order to reduce genotyping costs, much effort has been put on developing methods and software to impute genotypes at high-density chips from animals genotyped at low-density chips [11-15]. Accuracy of imputation may vary depending on the source of information being used to infer the genotypes and also on population structures. Hayes et al. [16] investigated the success of imputation from 5 k to 50 k genotypes in four sheep breeds and reported accuracies ranging from 71 to 80% depending on the breed. Erbe et al. [4] used the software BEAGLE [17] without pedigree information to impute genotypes at 800 k SNPs from dairy bulls genotyped at 50 k and reported accuracies of imputation (defined as the proportion of correctly imputed genotypes) ranging from 0.96 to 0.98 in Jersey and Hosltein cattle, respectively. Meuwissen and Goddard [18] applied a method for imputing whole-sequence genotypes on individuals genotyped at a low density panel and reported that 10% of the missing genotypes were erroneously imputed.

In principle, an increase in marker density should result in higher LD between the markers and the quantitative trait loci underlying a given trait, and consequently in more accurate genomic predictions. However, the advantage of using a high-density panel for GS compared to a low-density panel depends on which markers are included in the low-density panel. Such a formulation can be interpreted in terms of variable selection in a linear model, which has been a topic of frequent research aiming at reducing over-parameterisation in statistical models for GS [19,20], as well as making the implementation of a genomic breeding program more cost-effective [21]. Based on simulation analyses, Habier et al. [22] showed that low-density marker panels could be used in GS with a limited loss in accuracy compared to that achieved with high-density panels.

According to a study using dairy cattle data by Weigel et al. [21], moving from a set of 300 markers to a set of 2000 markers represented a gain in accuracy of ~30% or ~113%, depending on how the subsets of markers were selected (with largest effects or equally spaced). When moving from 2000 to 32 518 markers, gains in accuracy were only ~8% or ~13%. There is further empirical evidence that the relationship between gain in accuracy and increase in marker density tends to reach a plateau. VanRaden et al. [23] reported an average difference in accuracy of only 0.4% between predictions from a 50 k and a high-density (777 k) chip. As suggested by the results from VanRaden et al. [24], an increase in the number of animals in the training set should be more effective for improving the accuracy of genomic predictions than increasing the number of markers, especially when there is evidence that the benefit from increasing density tends to reach a plateau.

Many of the studies done with imputation so far have focused on the increase in density of markers panels through imputation and its impact on accuracy of genomic predictions. Results from Weigel et al. [25] in Jersey cattle indicated that if a suitable reference population genotyped with a 50 k chip is available, genotyping selection candidates with a 3 k instead of a 50 k chip and then imputing the remaining genotypes would result in a loss of predictive ability of only 5%. Dassonneville et al. [26] also studied the effect of genotyping selection candidates either with a 50 k or with a 3 k chip followed by imputation and reported losses in reliability ranging from 0.02 to 0.06 in Holstein cattle. Erbe et al. [4] used dairy cattle data to investigate the impact on the accuracy of genomic predictions of an increase in marker density from 50 k to 800 k through imputation, and reported an average gain in accuracy of 0.01 in Holsteins and 0.03 in Jersey cattle.

Imputation can be used to increase the number of markers. However, the benefit is expected to reach a plateau beyond a certain density. Imputation can also be used to increase the size of the training set with animals that were not genotyped at all. Cleveland et al. [27] investigated the impact of imputation on genomic predictions, and compared a training set of fully genotyped males and females with a training set in which only males were genotyped and females were imputed. An alternative and interesting analysis would be to compare the accuracy achieved in a training set with only genotyped males to that achieved with a training set containing the imputed females as well. A situation somewhat similar to that was investigated by Pszczola et al. [28], who compared a training set of genotyped bulls with a training set enlarged by imputed bulls. They used an additive relationship matrix relating genotyped to un-genotyped bulls to perform the imputation and reported an accuracy of imputation of 0.59, but the inclusion of the imputed bulls in training did not increase the accuracy of genomic prediction. This may be explained by the fact that the un-genotyped (imputed) bulls in their population had no offspring and the highest degree of relationship between them and the genotyped bulls was half-sib or parent-offspring. Imputation may be improved if the un-genotyped individuals to be imputed are defined in a specific design such that genotypes can be inferred with higher probabilities. For instance, imputation is likely to be more accurate when genotyped close relatives are available [29]. In some applications of GS, this may occur naturally. For example, when a training set is created for GS on traits that are expressed only in females, as for new traits in dairy cattle for which the cows’ phenotypes are difficult to measure (e.g. [30]) and/or for which accurate conventionally estimated breeding values of bulls are not yet available as an alternative response variable. In most livestock species, the number of males used for breeding is usually limited, thus when a reference population of males is in an advanced stage (as in the dairy industry, for example) most of the intensively used breeding males have probably been already genotyped. We consider a situation where a reference population of females is created and all or most of their sires and maternal grandsires have been already genotyped. In such cases, there is a considerable amount of family information available that can be used to try to infer the genotypes of the dams of these females. The configuration of the genotyped family members in this specific design should allow a much better quality of imputation of the un-genotyped dams than when performing imputation in a general framework on subjects from a pedigree with variable levels of relationships to the genotyped individuals. Although specific, this design is relevant since it will naturally arise in all future applications of genomic selection for new phenotypes.

The objectives of this work were: (1) to investigate the performance of two imputation methods for a completely un-genotyped dam, using the information on its genotyped family members and the mating partner plus the estimates of either allele or haplotype frequencies; (2) to investigate the effects of different population structures, levels of LD and distribution of allele frequencies on the success of imputation; and (3) to evaluate the impact of enlarging a training set with imputed dams on the accuracy of genomic predictions for different populations, levels of heritability (h^2^) of the trait under selection and sizes of training sets already available.

## Methods

### Imputation procedures

**Figure 1:**
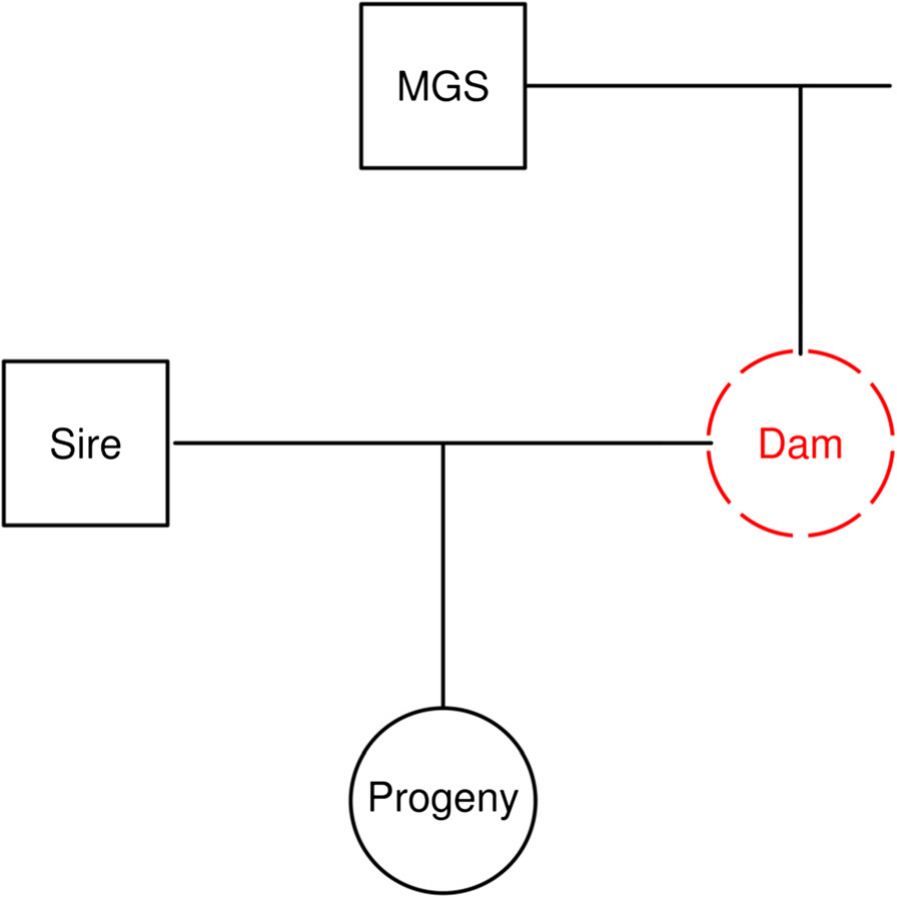
**Assumed family members with available genotypic information (black) used for imputing an un**-**genotyped dam (red).**

In the approach presented here, the aim was to impute the genotype of a completely un-genotyped dam. It is assumed that the genotypes of its sire, of one offspring and of this offspring’s sire are available. These three animals will be referred as MGS, Offspring and Sire. The situation is illustrated in Figure 1. Assume a bi-allelic locus with alleles coded as allele *1* and allele *2*. Let the frequency of allele *1* in the population be p and the frequency of allele *2* be q. In some cases, depending on the genotype configuration of the genotyped relatives and the mating partner and disregarding the probability of mutation, the genotype of the dam can be inferred unambiguously. For example, if the MGS is *11*, the Offspring is *12* and the Sire is *11*, then the dam must have inherited an allele *1* from the MGS and given an allele *2* to the Offspring. Therefore one can infer that the dam’s genotype is *12* with probability 1. In some other cases, inference about the dam’s genotype cannot be made unambiguously, but with a probability that is lower than 1.

The first imputation procedure (referred to as the Single_Step method) uses the information from the MGS, Sire and Offspring genotypes and allele frequencies to infer the dam’s genotypes for all loci, unambiguously or not. For each genotype configuration of the MGS, Offspring and Sire, each possible genotype of the dam can be assigned a probability, which in the ambiguous cases can be expressed as a function of the allele frequencies. These probabilities for each case were derived and are available in Additional file 1: Table S1. Calculation was done following Bayes’ theorem. Let the set of all three possible genotypes in a locus be $G=\left\{\begin{array}{ccc} 11 & 12 & 22 \end{array}\right\}$ and M, S, O and D be the genotypes of the MGS, Sire, Offspring and Dam. The posterior probability of the Dam’s genotype was then calculated as:

$$\begin{array}{l} P\left(D=G_{i}|M=G_{j}\cap S=G_{k}\cap O=G_{l}\right)=\\ \;=\frac{P\left(O=G_{l}|S=G_{k}\cap D=G_{i}\right)P\left(D=G_{i}|M=G_{j}\right)}{\sum_{m=1}^{3}P\left(O=G_{l}|S=G_{k}\cap D=G_{m}\right)P\left(D=G_{m}|M=G_{j}\right)} \end{array}$$

The second imputation procedure is done in two stages and therefore will be referred to as the Two_Step method. In a first step, only the Dam genotypes that can be inferred with probability 1 are assigned (see Additional file 1: Table S1) and whenever the probability is lower than 1, the Dam genotype is set to missing. In a second step, the genotyping data from the Dam, containing assigned and missing genotypes, are combined with all available genotyping data from the MGS, Offspring and Sire, and missing genotypes are filled in using LD information. The second step was carried out using the software fastPHASE [31] for haplotype reconstruction and inference of missing genotypes.

To assess the efficiency of the two methods described above, imputation of Dam genotypes was also performed using two currently available imputation programs: findhap.f90 Version 2 [15] and AlphaImpute Beta 1.16 [29].

### Simulation

For the comparison of imputation methods, genomic data were simulated using the software QMSim [32]. The simulated genome consisted of one chromosome of 100 cM, on which 2000 bi-allelic markers (coded as alleles *1* and *2*) were randomly allocated. Marker allele frequencies in the first historical generation were set equal to 0.5 and the mutation rate was set to 2.5e-5. In order to generate different genomic structures that may influence the success of imputation, four populations were simulated, which differed in the level of LD and the presence or absence of selection. The increase in the level of LD desired for two of the populations was induced by simulating a bottleneck in the historical population. Therefore, the four scenarios were created as follows: no bottleneck and no selection (LowLD_NoSel), no bottleneck and selection (LowLD_Sel), bottleneck and no selection (HighLD_NoSel), and bottleneck and selection (HighLD_Sel). For each of the four scenarios, 10 replicates were simulated.

To generate a minimum level of LD for the two scenarios without bottleneck, a historical population of 4000 animals was mated at random for 1600 discrete generations, without selection, without migration and with an equal number of animals from both genders. Then the population size was increased to 4040 in the following 20 generations and kept at a constant size for 20 additional generations. For the two scenarios with bottleneck, the historical population was initially set to 2000 animals and mated at random for 2500 generations. After this, a bottleneck was simulated by gradually decreasing the population size to 200 animals over the following 70 generations; these 200 animals were further mated at random for 10 generations. The population size was then gradually expanded from 200 to 4040 animals within the next 20 generations, and remained at a size of 4040 for 20 additional generations. In all four scenarios, population size was 4040 in the last historical generation, which included 40 males.

Starting with the 4000 female and 40 male founders from the last historical generation, 10 additional generations were simulated to form the recent population. In the recent population, the proportion of male offspring was 0.5, litter size was 1, a random mating design was applied and replacement ratios for sires and dams were 0.5 and 0.25, respectively. These parameters were common to all four scenarios. For the two scenarios without selection, a random selection design was used and the culling design was based on the age of the animal. For the two scenarios with selection, both selection and culling designs were based on estimated breeding values (EBV). These EBV were obtained by solving Henderson’s mixed model equations [33] using pedigree information and phenotypic records from a trait with h^2^ = 0.20. Since the proportions of female and male offspring were identical, the last generation of the recent population contained 2000 female offspring. Genotype imputation was then performed on the dams of these 2000 female offspring from the last generation.

To investigate the impact of imputation on the accuracy of genomic predictions, the size of the training set used for SNP effect estimation is a relevant parameter. For that purpose, the same simulation procedures described above for the four scenarios were applied again in another simulation, in which a larger population was generated at the end. Instead of using a size of 4040 for the last historical generation, the number of female founders was set to 32 000 so that 16 000 female offspring in the last generation were available for the imputation of their dams. As above, 10 replicates of each scenario were simulated for the larger populations.

### Assessment of LD in the simulated populations

Outputs from QMSim included information about the paternal and maternal alleles of each locus, which allowed the determination of linkage phase and the calculation of haplotype frequencies. The level of LD in the four simulated scenarios could then be assessed by calculating the squared correlation coefficient (r^2^) between each pair of markers in the last generation. To minimize the influence of the minor allele frequency (MAF) on the measure of LD, r^2^ values were computed only for pairs of markers with a MAF greater than 0.05. The decay of LD with increasing inter-marker distances was also assessed by calculating the mean r^2^ within bins of inter-marker distances.

### Prediction of genomic breeding values

The impact of the imputation of Dam genotypes on the accuracy of genomic predictions was investigated for the imputation method with the best performance. For that purpose, the simulated data sets with 16 000 female offspring in the last generation were used. Four different sizes of training sets to estimate marker effects were created by splitting each replicate into subsets containing 2000, 4000, 8000 and 16 000 female offspring. From each subset, 90% of the animals were assigned to the training set and the remaining 10% to the validation set. Accuracy of genomic prediction was then assessed by cross-validation, i.e. marker effects were estimated with data from animals in the training set and used to predict genomic breeding values of animals in the validation set. The sizes of the training sets containing only genotyped animals (TS) were 1800, 3600, 7200 and 14 400. Training sets augmented (TSA) with imputed Dams were created, resulting in training sets of 3800, 7600, 15 200 and 30 400 animals. The impact of imputation was evaluated by comparing the accuracies of genomic predictions using TS or the corresponding TSA. To generate different levels of heritability (h^2^), different magnitudes of residual terms were added to the simulated true breeding values, generating phenotypic values representing ten levels of h^2^, ranging from 0.05 to 0.5 in steps of 0.05. For each size of training set and each h^2^, allele substitution effects of every locus on the simulated phenotypes were fitted in a multiple random regression model similar to the GBLUP method of Meuwissen et al. [1]. Estimated SNP effects were calculated from the following mixed model equations:

$$\left[\begin{array}{c} \hat{\mu }\\ \hat{\alpha } \end{array}\right]={\left[\begin{array}{cc} \iota^{t}\iota & \iota^{t}X\\ X^{t}\iota & X^{t}X+I\phi \end{array}\right]}^{-1}\left[\begin{array}{c} \iota^{t}y\\ X^{t}y \end{array}\right]$$

where μ is an overall mean; **α** is the vector of allele substitution effects; **ι** is a vector of ones, of order equal to the number of animals in the training set; **X** is the matrix of SNP genotypes, coded as the number of copies (or dosage) of allele *2*, of the animals in the training set; **y** is the vector of phenotypes; **I** is an identity matrix of order equal to the number of markers and ϕ is an assumed ratio of residual to marker variances. This ratio of variances was calculated using the simulated h^2^ values and assuming a marker variance equal to the additive variance divided by the number of markers. For each scenario and replicate, only markers with a MAF greater than 0.05 were used in the estimation of SNP effects. Genomic breeding values were then predicted as $\mathrm{GEBV}=\hat{\mu }\iota +Z\hat{\alpha }$, where **Z** is the matrix of SNP genotypes, coded as the number of copies of allele *2*, of the animals in the validation set. Accuracy of genomic evaluation was calculated as the correlation between GEBV and the simulated true breeding values of the animals in the validation set.

## Results and discussion

### LD and distribution of allele frequencies in the simulated populations

An overview of the level and the decay of LD with inter-marker distance for the four simulated populations used to compare the imputation methods is presented in Table 1. Bins of distance are expressed in kb assuming that one Mb is equivalent to one cM. As intended, the level of LD in the scenarios simulated with a bottleneck in the historical population (HighLD_) was higher than in the scenarios without bottleneck (LowLD_). Selection was also a factor that increased the overall level of LD, and even more strongly the extent of LD over larger inter-marker distances. In the scenarios without selection, LD decreased much more rapidly than in the scenarios with selection. At an inter-marker distance of 200–500 kb, the mean r^2^ was less than one third of the mean r^2^ at an inter-marker distance smaller than 25 kb, whilst in the scenarios with selection it was still more than a half of that (Table 1). Plots of all pair-wise values of r^2^ against inter-marker distance for all replicates of the four scenarios are provided in Additional file 2: Figure S1.

**Table 1:** **Mean linkage disequilibrium** (**r**^**2**^) **within different inter**-**marker distances in the simulated populations used for the comparison of imputation methods**

**Scenario**	**Inter-marker distance (kb)**					
	**<25**	**25-50**	**50-75**	**75-120**	**120-200**	**200-500**
LowLD_NoSel	0.15	0.13	0.12	0.10	0.08	0.05
LowLD_Sel	0.28	0.26	0.25	0.23	0.21	0.18
HighLD_NoSel	0.35	0.29	0.25	0.21	0.17	0.11
HighLD_Sel	0.48	0.43	0.37	0.34	0.30	0.24

Values are means across 10 replicates.

The different population structures simulated in the four scenarios not only affected the pattern of LD, but also caused different shapes of the distribution of allele frequencies. Histograms of the frequencies of allele *2* for all replicates of the four scenarios are provided in Additional file 2: Figure S2. In the LowLD_NoSel scenario (the one with the lowest level of LD), the distribution of allele frequencies was bell-shaped, with a much higher frequency of markers with intermediate allele frequencies compared to markers with extreme allele frequencies. In the HighLD_NoSel scenario, the distribution was more uniform, with a slightly higher frequency of markers with extreme allele frequencies. Selection caused a higher frequency of markers with extreme allele frequencies, especially in the scenario HighLD_Sel. Variability in the distributions across replicates was large in the scenarios LowLD_Sel and HighLD_Sel, whilst a very uniform pattern was observed in the LowLD_NoSel and HighLD_NoSel scenarios.

The level of LD directly affects the performance of the Two_Step method, since information on haplotype frequencies is used by fastPHASE to impute the missing genotypes. The Single_Step method does not use LD information but its performance will be affected by the different shapes of the distribution of allele frequencies, since the genotypes of markers with more extreme allele frequencies are easier to impute.

### Quality of imputation between scenarios

The success rates of imputation (defined as the percentage of correctly imputed genotypes) for each imputation method and each scenario, averaged across replicates, are presented in Table 2. Mean success rates ranged from 0.70 to 0.85 in the Single_Step and from 0.60 to 0.80 in the Two_Step method. Pszczola et al. [28] simulated a dairy cattle population and imputed genotypes on completely un-genotyped bulls in a mixed model approach using the additive relationship matrix, and reported an accuracy of imputation of 0.58. In their approach, imputation was performed using only information from related genotyped animals, and the highest degree of relationship between genotyped and un-genotyped animals in their simulated population was half-sib or parent-offspring. Cleveland et al. [27] investigated genotype imputation on dams in the training set in a simulated population. Their method used segregation analysis and information on haplotype frequencies, and they reported a success rate of 69% when dams were completely un-genotyped. Nevertheless, the available family information for each dam in their training dataset did not exactly correspond to the situation considered in our design, as shown in Figure 1.

**Table 2:** Percentage of correctly imputed genotypes of the Dams for two imputation methods

**Imputation method**	**Scenario**			
	**LowLD_NoSel**	**LowLD_Sel**	**HighLD_NoSel**	**HighLD_Sel**
Single_Step	0.70 ± 0.003	0.77 ± 0.045	0.81 ± 0.005	0.85 ± 0.019
Two_Step	0.60 ± 0.004	0.71 ± 0.056	0.75 ± 0.005	0.80 ± 0.021

Values are the means ± standard deviations across 10 replicates.

**Figure 2:**
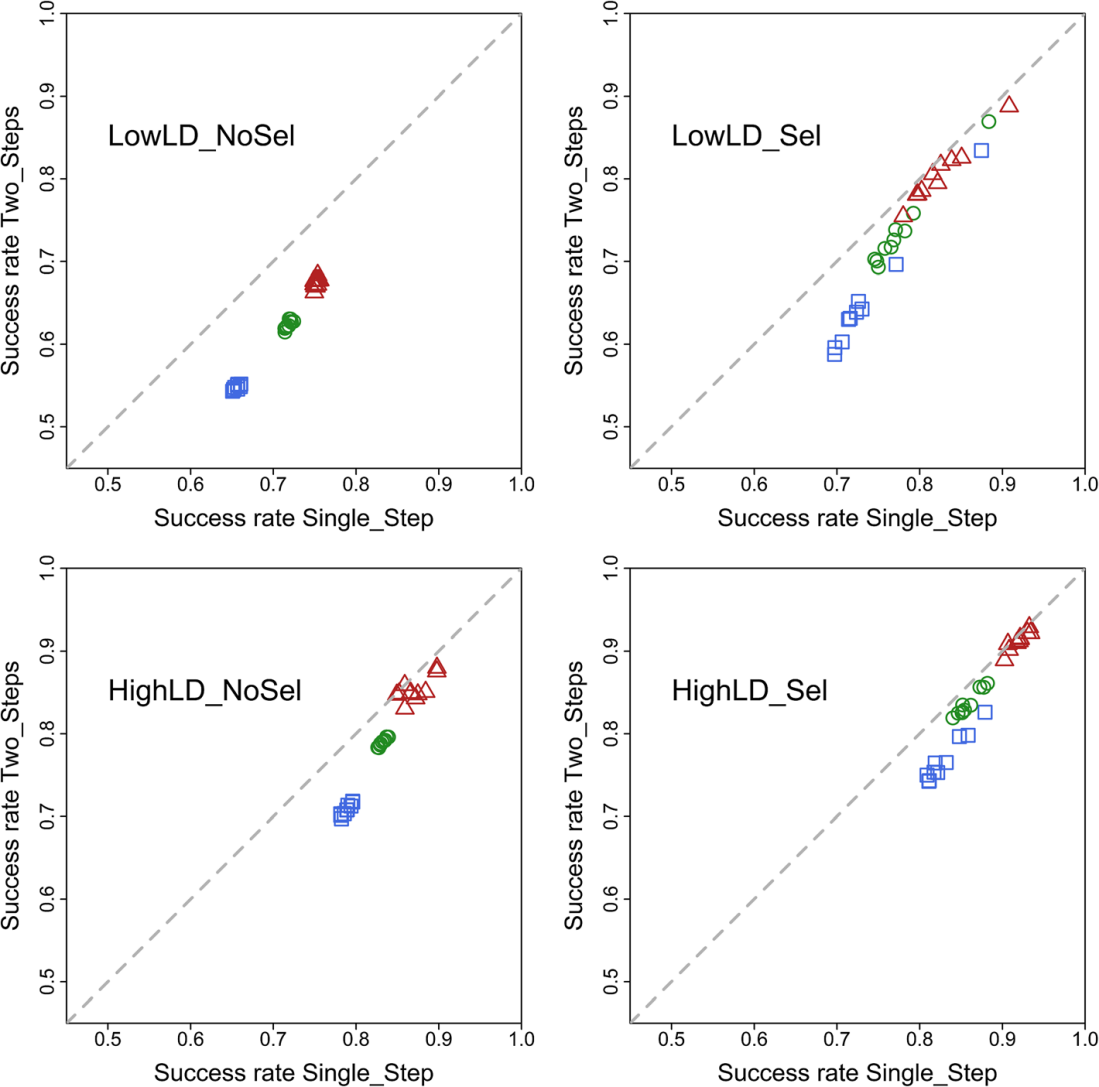
**Average proportion of correctly imputed genotypes within each class of Dams**, **from both imputation methods for each simulated population structure.** Each data point is the mean percentage within Dam class in a given replicate. Classes correspond to number of genotypes unambiguously inferred: blue square (lower than 100), green circle (between 100 and 300) and red triangle (greater than 300).

Overall, the Single_Step method performed better than the Two_Step method for the four simulated scenarios. As expected, the quality of imputation with the Two_Step method increased with higher levels of LD. A similar trend was observed with the Single_Step method. Although LD information is not directly used in the Single_Step method, its performance was influenced by the level of LD since the simulated populations with a higher level of LD presented distributions of allele frequencies with greater densities at more extreme allele frequencies (see Additional file 2: Figure S2). At any given locus, the quality of imputation with the Single_Step method depends on the probability with which the Dam’s genotype can be imputed. As can be seen in Additional file 1: Table S1, this probability is a function of the allele frequencies at the locus and the genotype configuration of the MGS, Sire and Offspring. The larger the number of loci for which the genotype can be imputed with a probability of one, the higher is the expected proportion of correctly assigned genotypes to a given Dam. The number of unambiguously assigned genotypes is expected to have an even greater impact on the quality of imputation with the Two_Step method. This number defines how many loci can be assigned in the first step, which is the only step where information on genotypes of the MGS, Sire and Offspring is used. In order to better assess the dependency of the relative success rate of each method on the number of unambiguously assigned genotypes, Dams were grouped in three classes according to the number of loci imputed with a probability of one: less than 100 loci, between 100 and 300 loci, and more than 300 loci. Distributions of the number of unambiguously imputed loci per Dam for all replicates of the four scenarios are provided in Additional file 2: Figure S3. The average success rate within each group for each replicate is shown in Figure 2 for each of the four simulated scenarios. As expected, the larger the number of genotypes that could be inferred with a probability of one, the higher the quality of imputation for both methods. A similar relationship between success rate and proportion of alleles inferred without ambiguity was also reported by Hickey et al. [29]. In our study, this effect was more pronounced with the Two_Step method because Dams with no or very few assigned genotypes in the first step move to the second step with all or almost all genotypes set to missing. Imputation in the second step is then based exclusively or mostly on haplotype frequencies alone. Weigel et al. [34] masked different proportions of SNP genotypes in some animals from a data set of Jersey cattle and used fastPHASE to impute them. They reported mean success rates ranging from 0.66 to 0.72 when 1 to 2% of genotypes were available. Mean success rates increased to 0.75 to 0.88 when 5 to 10% of genotypes were available and to 0.90 to 0.94 when 20% of the genotypes were available. These success rates are similar to those observed in our HighLD_ scenarios (Figure 2). The average numbers (percentage) of unambiguous loci per Dam were 159.3 (8.0%), 154.5 (7.7%), 107.6 (5.4%) and 99.9 (5.0%) for LowLD_NoSel, LowLD_Sel, HighLD_NoSel and HighLD_Sel, respectively. Nevertheless, these 5 to 8% of genotypes inferred unambiguously that were made available to fastPHASE in the second step are in general different SNP between Dams. The average success rate with the Single_Step method was greater than with the Two_Step method for all groups of Dams. The difference in performance of the two methods was larger for lower levels of LD and for a smaller number of loci that could be inferred in the first step of the Two_Step method (i.e., the number of unambiguous cases). The higher the level of LD, the closer the performance of the two methods, especially for the group of Dams with more than 300 loci inferred in the first step. This illustrates a higher dependency of the Two_Step method on the level of LD. In the scenario with the highest level of LD (HighLD_Sel), the success rates of both methods were almost the same for the group of Dams that had more than 300 unambiguously inferred genotypes. Even for the simulated scenario with the highest level of LD, the Two_Step method did not outperform the Single_Step method. This indicates that, for the specific design considered here (Figure 1), the probabilities derived from family information used in the Single_Step were more useful to infer genotypes at ambiguous loci than LD information. For the unambiguous loci, the two methods are exactly the same. A difference is observed only when all genotype probabilities at the locus are lower than 1. Depending on the MAF at the locus and the genotypic configuration of the MGS, Sire and Offspring, the most probable genotype from Single_Step method may still be associated with a posterior probability that is higher than could be inferred based on haplotype frequencies alone.

The average success rate for both methods of imputation for Dams that had more than 300 unambiguously inferred genotypes in the HighLD_Sel scenario was ~0.92 (Figure 2). This proportion is similar to what one would expect to achieve when moving from a low density to a higher density panel of markers e.g. [35,36]. In both approaches described here, this level of success rate could be achieved for completely un-genotyped Dams.

The Two_Step method could be compared to imputation from low to high density (e.g., 3k to 50k), in which first a ‘low density chip’ is built based on the unambiguous cases and then the rest is filled in with LD information. However, three main differences must be pointed out: (i) the Two_Step method starts from completely un-genotyped animals; (ii) after the first step, Dams have genotypes for a ‘low density chip’ but with a different chip for each Dam and not a set of evenly spaced markers common to all Dams; and (iii) information on the genotyped relatives is used only in the first step, which means that after the ‘low density chip’ is built the only information available for imputation is LD, whereas in a low to high density approach, one would still have the possibility of using family information. Obviously, if a low density panel of SNP was also available for these Dams, the average success rate would be even greater, but at the cost of genotyping the Dams for the low density chip. Inspection of the number of genotypes which can be imputed unambiguously may provide an approximate estimate of the expected success rate that may be achieved by imputation. Such an estimate could then be used as an aid to choose the Dams to be genotyped with a low density chip. In the case of a group of Dams, for which say 10 or 15% of the loci can be unambiguously inferred from family information alone, one could choose to leave them completely un-genotyped and do imputation with the Single_Step method. Knowledge about the population structure under consideration (e.g., level of LD and distribution of allele frequencies) would also be required in such a decision process. In order to account for that, simple experiments (e.g., genotyping and imputing a small number of Dams) could be conducted to empirically estimate the expected success rate for Dams with a given number of loci inferred with a probability of one.

One aspect of the imputation procedures proposed here is that genotypic information is assumed to be available on a specific set of animals (Figure 1), including one offspring. These methods can, however, be extended to situations in which a number of genotyped offspring are available, which should considerably improve the quality of imputation. Such improvement would be expected for both methods, since a larger number of offspring would most likely result in a larger number of unambiguous cases.

### Comparison with available software

According to the algorithm described in Additional file 1: Table S1, the genotype at each locus can be assigned to be *11*, *12* or *22*. For genomic selection purposes and according to how marker genotypes are modelled (i.e. as a covariate representing the number of copies of a given allele), genotypes at each locus can also be assigned a continuous value within the range 0–2. Instead of the number of copies of allele *2*, genotypes are defined as allele *2* dosage. This definition avoids loss of information caused by rounding the genotype to one of the three classes. Allele dosages for each genotype configuration of the MGS, Sire and Offspring as a function of allele frequencies were derived and are provided in Additional file 1: Table S2 (this version will be further referred to as the Single_Step*). Since our aim was to compare the results with those obtained with other software, which, in some cases, construct imputed genotypes as the sum of allele probabilities (e.g., AlphaImpute), genotypes were imputed as allele dosage. Quality of imputation in this case was assessed via the correlation coefficient between real and imputed allele dosages, and will be further referred here as the accuracy of imputation. Evaluating the quality of imputation in this way also has some advantages compared to the success rate, for the reasons pointed out by Hickey et al. [37]. Accuracy of imputation from all methods and software are presented in Table 3. Imputing an allele dosage instead of assigning the most probable genotype resulted in a gain in accuracy from 2.3% (HighLD_Sel) to 6.4% (LowLD_NoSel).

**Table 3:** Correlation between true and imputed genotypes from different imputation methods and programs

**Imputation method**	**Scenario**			
	**LowLD_NoSel**	**LowLD_Sel**	**HighLD_NoSel**	**HighLD_Sel**
Single_Step	0.76 ± 0.003	0.83 ± 0.038	0.88 ± 0.004	0.90 ± 0.013
Single_Step*	0.81 ± 0.003	0.86 ± 0.028	0.90 ± 0.003	0.93 ± 0.009
Two_Step	0.57 ± 0.008	0.74 ± 0.066	0.80 ± 0.006	0.85 ± 0.021
findhap.f90	0.52 ± 0.006	0.69 ± 0.065	0.74 ± 0.006	0.82 ± 0.030
AlphaImpute	0.83 ± 0.003	0.87 ± 0.024	0.86 ± 0.004	0.89 ± 0.010

Values are the means ± standard deviations across 10 replicates.

Imputation accuracies from findhap.f90 were lower than accuracies from Single_Step* and Two_Step. The algorithm implemented in findhap.f90 is a combination of pedigree haplotyping and population haplotyping. Our results indicate that the amount of genotyping information available in the situation considered here (i.e., MGS, Sire and Offspring) seemed to be insufficient for the pedigree haplotyping algorithm to satisfactorily impute a completely un-genotyped Dam. Many other studies reporting performance results from findhap.f90 applied the program with the main purpose of imputing genotypes from low to high density chips [15,35,36]. In such cases, findhap.f90 can take more advantage of the population haplotyping algorithm because of the observed genotypes from the low density chip and may perform imputation with an accuracy greater than 0.95. To resemble an application with a small chip, we performed another series of imputation runs with findhap.f90, in which Dams had genotypes for 125 evenly spaced markers. Average imputation accuracies from findhap.f90 when moving from the sparse (125) to the dense (2000) set of markers were 0.96 (LowLD_NoSel), 0.98 (LowLD_Sel), 0.97 (HighLD_NoSel) and 0.98 (HighLD_Sel). These numbers are not comparable to the results in Table 3. They are rather used to illustrate the magnitude of accuracy expected when imputation is applied to move from low to high density chips, which also indicates a strong dependency of the performance of findhap.f90 on the number of unambiguously imputed loci.

Accuracies of imputation from AlphaImpute were higher than from the Two_Step method, especially in the LowLD scenarios. In some cases, although the complete genotypes cannot be inferred unambiguously, one can at least be sure about the presence of one of the alleles. This piece of information is neglected by the Two_Step method, since when moving from the first to the second step, the only information available for haplotype reconstruction are the unambiguous genotypes. An improvement in imputation accuracy from the Two_Step method could be achieved if known alleles were also taken into account in the haplotyping step. This information seems to be more efficiently used by the algorithm implemented in AlphaImpute, which is a combination of long-range phasing and haplotype library imputation. Results from AlphaImpute were similar to results obtained with Single_Step*. In the LowLD scenarios, AlphaImpute performed better and in the HighLD scenarios, results from Single_Step* were better. The strength of AlphaImpute is its flexibility, since it can handle different levels of relationship between the surrogate and the genotyped animals. The strength of the Single_Step* method is its simplicity and ease of programming, which enables very fast imputation. Since the difference in performance was smaller in the LowLD than in the HighLD scenarios and the intended application was for the specific situation considered here, Single_Step* was the method of choice to investigate the impact of imputation on accuracy of genomic predictions.

### Impact on the accuracy of genomic breeding values

**Figure 3:**
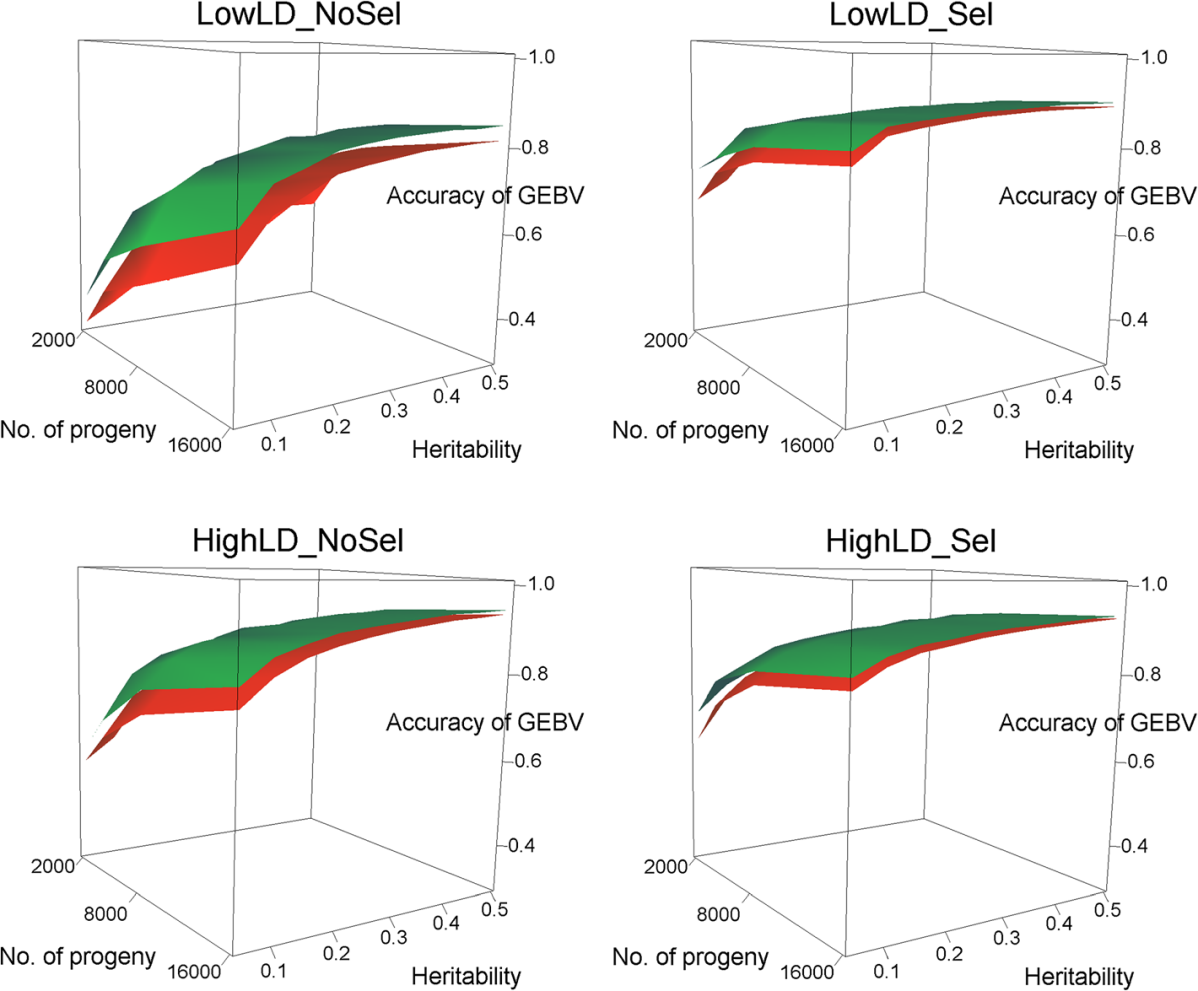
**Accuracies of genomic prediction for different values of trait h**^**2**^, **number of female progeny in the last generation and population structure.** Red surfaces represent accuracies obtained with TS (90% of the progeny in the training set) and green surfaces represent accuracies obtained with TSA (90% of the progeny + the imputed Dams).

An overview of the level and the decay of LD with inter-marker distance for each of the four simulated populations used to investigate the impact of imputation on the accuracy of genomic predictions is presented in Table 4. Trends are quite similar to those in Table 1. Augmentation of TS into TSA was done by imputing Dams’ genotypes using the Single_Step* method. Correlations between GEBV and true breeding values in the validation set using either TS or TSA to estimate SNP effects are shown in Figure 3 for the four simulated scenarios and for different h^2^ and numbers of offspring. As a general trend, accuracy of genomic predictions increased with increasing h^2^ and increasing sizes of training sets. This is consistent with the formula proposed by Daetwyler et al. [38], in which the expected accuracy of genomic prediction with GBLUP is calculated as a function of the number of animals in the training set, h^2^ and the number of independent chromosome segments. The simulated population structure also had an impact on prediction accuracy. As expected, accuracies on average increased with increasing levels of LD observed from scenario LowLD_NoSel to scenario HighLD_Sel. These differences between scenarios are also consistent with the formula of Daetwyler et al. [38], in which they suggest calculating the number of independent chromosome segments as a function of the genome length and the effective population size, following Goddard [39]. These parameters should in turn vary for different genome and population structures, with different levels of LD and distributions of allele frequencies. All correlation coefficients between the GEBV and true breeding values in the validation set obtained with TS and TSA for all simulated scenarios, h^2^ and numbers of offspring are provided in Additional file 1: Table S3. Although the imputation accuracies were smaller for Single_Step than for Single_Step*, the accuracies of genomic breeding values using either method were almost identical (differences were smaller than 1% on average) and show exactly the same pattern as for Single_Step* (results not shown).

**Table 4:** **Mean linkage disequilibrium** (**r**^**2**^) **within different inter**-**marker distances in the simulated populations used for the comparison of the accuracy of genomic predictions with and without imputation**

**Scenario**	**Inter-marker distance (kb)**					
	**<25**	**25-50**	**50-75**	**75-120**	**120-200**	**200-500**
LowLD_NoSel	0.14	0.12	0.10	0.08	0.06	0.03
LowLD_Sel	0.21	0.19	0.18	0.16	0.14	0.12
HighLD_NoSel	0.36	0.30	0.26	0.22	0.17	0.11
HighLD_Sel	0.43	0.37	0.32	0.29	0.24	0.18

Values are the means across 10 replicates.

An increase in the accuracy of GEBV was observed when using TSA instead of TS, which demonstrates that enlarging a training set with imputed Dams represents an advantage. The extent of this advantage differed between the different population structures simulated. In the LowLD_NoSel scenario, the gain in accuracy, expressed as percentage of the accuracy with TS, ranged from 3.7% to 37.1%. The benefit of incorporating imputed Dams in the training set was overall larger for this scenario, despite the fact that with this scenario genotype imputation was performed with the poorest quality. In the other three scenarios, the maximum gains were 11.1% (LowLD_Sel), 15.3% (HighLD_NoSel) and 11.9% (HighLD_Sel), and the minimum gains were close to zero. Because imputation is not perfect, the increase in accuracy obtained with TSA was generally lower than what could be achieved by enlarging TS with another set of genotyped offspring. For each of the four scenarios, we compared the increase in accuracy obtained when: (1) enlarging TS by doubling the number of genotyped offspring; or (2) enlarging TS with imputed Dams. For example, in the LowLD_NoSel scenario with an h^2^ of 0.05, moving from a TS of 1800 to a TS of 3600 offspring gave a gain in accuracy of 32% (from 0.31 to 0.41). Adding the 2000 imputed Dams to a TS of 1800 offspring (i.e., a TSA of 3800 animals) gave a gain in accuracy of 23% (from 0.31 to 0.38), which is 72% of the gain in the first case and reflects the fact that the proportion of correctly imputed genotypes of Dams is lower than 1. On average, across all h^2^ and numbers of offspring, the gain in accuracy in the second case was 93% (LowLD_NoSel), 62% (LowLD_Sel), 78% (HighLD_NoSel) and 63% (HighLD_Sel) of the gain in accuracy obtained in the first case. The first case would require doubling the costs by genotyping another set of offspring, whereas in the second case, no additional costs for genotyping are needed. If there is funding available for genotyping more animals, then increasing the size of the training set with genotyped animals should improve the accuracy of genomic predictions more. Different strategies can be used to genotype more animals, e.g. genotyping for a low density chip the Dams with very few loci for which imputation can be unambiguously made, as pointed out in the previous section. Nevertheless, according to our results, even if all available funding for genotyping has been spent, there is still room for an additional improvement in genomic predictions by enlarging TS with imputed Dams.

The magnitude of the gain in accuracy when moving from TS to TSA varied not only between scenarios but also for different values of h^2^ and numbers of offspring already available in TS. The effects of h^2^, number of offspring and simulated scenario on the difference between accuracies obtained with TS and TSA were all significant (P < 0.001). Pszczola et al. [28] added 1000 imputed bulls to a training set of 1000 genotyped bulls and did not find any significant increase in accuracy of genomic predictions. The authors attributed their finding to the low accuracy of imputation in their study (0.58). Nevertheless, Pszczola et al. [28] reported a trend of increasing difference in accuracy with decreasing h^2^, which is consistent with our results. The population of Pszczola et al. [28] was simulated to resemble a dairy cattle population with a considerably high level of LD (average r^2^ of 0.41 between adjacent markers, which were on average 0.13 cM apart). This level of LD is higher than that observed in our scenario with the highest LD (HighLD_Sel), in which the increase in accuracy of genomic predictions was overall the lowest in our study. This agrees with our indication that the impact of enlarging a reference population with imputed individuals in terms of accuracy of genomic prediction depends on the population structure under consideration.

Generally, the larger the accuracy already obtained with TS, the lower is the increase in accuracy achieved with TSA. Regression analyses of the percentage increase in accuracy obtained with TSA against the accuracy already obtained with TS across all h^2^ and numbers of offspring for the four scenarios were performed. Results fitted a negative linear relationship well, with coefficients of determination of 0.80, 0.88, 0.68 and 0.85 for scenarios LowLD_NoSel, LowLD_Sel, HighLD_NoSel and HighLD_Sel (Additional file 2: Figure S4). This pattern was not only observed when moving from TS to TSA, but also when moving from a smaller to a larger TS. This can also be seen in the shapes of the surfaces presented in Figure 3, in which the increase in accuracy resulting from an increase in either h^2^ or the number of offspring tends to reach a plateau.

## Conclusions

Genotypes of a dam’s sire, one offspring and this offspring’s sire, as well as estimates of marker allele frequencies were used to impute genotypes of dams with an accuracy, i.e. the correlation between observed and imputed genotypes, ranging from 0.81 to 0.93. Accuracy of imputation was higher in populations with higher levels of LD and with distributions of allele frequencies containing a larger proportion of markers with more extreme allele frequencies.

Overall, inclusion of imputed dams in the training set increased genomic predictions, up to 37%. The impact of enlarging the training set with imputed dams on the accuracy of genomic predictions depends on the heritability of the trait, on the number of animals in the already available training set, and on the population structure.

Besides being useful for reducing costs of genotyping by imputing high-density panels on animals genotyped with low-density panels, imputation can also be used to achieve an extra increase in accuracy of genomic predictions by enlarging the training set with completely un-genotyped dams. This strategy is particularly useful for populations with low levels of LD, for genomic selection on traits with low h^2^, and for species or breeds for which the reference population size is limited.

## Competing interests

The authors declare that they have no competing interest.

## Authors’ contributions

ECGP and MWD developed the Single_Step and Two_Step methods, wrote all computer programs, and shared the running of computations. ECGP wrote the manuscript. SK and HHS contributed in conceiving the ideas, setting up the study, and assisted in writing the manuscript. All authors have read and approved the final manuscript.

## Supplementary Material

Additional file 1: Table S1 Probabilities of each Dam genotype at a SNP, given genotypes of relatives (maternal grandsire (MGS), Sire, and one Offspring) and frequencies of alleles 1 (p) and 2 (q), and decision algorithm for assigning the imputed Dam genotype. **Table S2.** Imputed allele dosage on each Dam given the genotype configuration of relatives and the frequencies of allele 1 (p) and 2 (q). **Table S3.** Correlation between true and genomic estimated breeding values in the validation set obtained when estimating SNP effects with Offspring only (TS) or with an augmented training set including imputed Dams (TSA). Click here for file

Additional file 2: Figure S1 Pair-wise values of r^2^ against inter-marker distance for all replicates of the four scenarios. **Figure S2.** Histograms of the frequencies of allele *2* for all replicates of the four scenarios. **Figure S3.** Distributions of the number of unambiguously imputed loci per Dam for all replicates of the four scenarios. **Figure S4.** Description: Regression analyses of the percentage increase in accuracy obtained with TSA against the accuracy already obtained with TS across all h^2^ and numbers of offspring for the four scenarios. Click here for file
