# Supplementary material for: Enlarging a training set for genomic selection by imputation of un-genotyped animals in populations of varying genetic architecture
Source: Genet Sel Evol. 2013 Apr 26;45(1):12. doi: 10.1186/1297-9686-45-12 (PMC3652763; doi:10.1186/1297-9686-45-12)
Supplement: Additional file 1: Table S1 — Probabilities of each Dam genotype at a SNP, given genotypes of relatives (maternal grandsire (MGS), Sire, and one Offspring) and frequencies of alleles 1 (p) and 2 (q), and decision algorithm for assigning the imputed Dam genotype. Table S2. Imputed allele dosage on each Dam given the genotype configuration of relatives and the frequencies of allele 1 (p) and 2 (q). Table S3. Correlation between true and genomic estimated breeding values in the validation set obtained when estimating SNP effects with Offspring only (TS) or with an augmented training set including imputed Dams (TSA). [file 1297-9686-45-12-S1.pdf]

Table S1. Probabilities of each Dam genotype at a SNP, given genotypes of relatives (maternal grandsire (MGS), Sire, and one Offspring) and frequencies of alleles *1* (p) and *2* (q), and decision algorithm for assigning the imputed Dam genotype.

MGS	Sire	Offspring	Probability of Dam genotype			Imputed genotype
			11	*12*	*22*	
11	*11*	*11*	$\frac{p}{p+0.5q}$	$\frac{0.5q}{p+0.5q}$	0	*11* if p > q/2 and *12* otherwise
11	*11*	*12*	0	1	0	*12*
11	*12*	*11*	$\frac{p}{p+0.5q}$	$\frac{0.5q}{p+0.5q}$	0	*11* if p > q/2 and *12* otherwise
11	*12*	*12*	p	q	0	*11* if p > q and *12* otherwise
11	*12*	*22*	0	1	0	*12*
11	*22*	*12*	$\frac{p}{p+0.5q}$	$\frac{0.5q}{p+0.5q}$	0	*11* if p > q/2 and *12* otherwise
11	*22*	*22*	0	1	0	*12*
12	*11*	*11*	$\frac{p}{p+0.5}$	$\frac{0.5}{p+0.5}$	0	*11* if p > q and *12* otherwise
12	*11*	*12*	0	$\frac{0.5}{q+0.5}$	$\frac{q}{q+0.5}$	*22* if q > p and *12* otherwise
12	*12*	*11*	$\frac{p}{p+0.5}$	$\frac{0.5}{p+0.5}$	0	*11* if p > q and *12* otherwise
12	*12*	*12*	0.5p	0.5	0.5q	*12*
12	*12*	*22*	0	$\frac{0.5}{q+0.5}$	$\frac{q}{q+0.5}$	*22* if q > p and *12* otherwise
12	*22*	*12*	$\frac{p}{p+0.5}$	$\frac{0.5}{p+0.5}$	0	*11* if p > q and *12* otherwise
12	*22*	*22*	0	$\frac{0.5}{q+0.5}$	$\frac{q}{q+0.5}$	*22* if q > p and *12* otherwise

22	*11*	*11*	0	1	0	*12*
22	*11*	*12*	0	$\frac{0.5p}{q+0.5p}$	$\frac{q}{q+0.5p}$	*22* if q > p/2 and *12* otherwise
22	*12*	*11*	0	1	0	*12*
22	*12*	*12*	0	p	q	*22* if q > p and *12* otherwise
22	*12*	*22*	0	$\frac{0.5p}{q+0.5p}$	$\frac{q}{q+0.5p}$	*22* if q > p/2 and *12* otherwise
22	*22*	*12*	0	1	0	*12*
22	*22*	*22*	0	$\frac{0.5p}{q+0.5p}$	$\frac{q}{q+0.5p}$	*22* if q > p/2 and *12* otherwise

Table S2. Imputed allele dosage on each Dam given the genotype configuration of relatives and the frequencies of allele *1* (p) and *2* (q).

MGS	Sire	Offspring	Probability of dam genotype			Imputed allele dosage
			11	*12*	*22*	0*P(11)+1*P(12)+2*P(22)
11	*11*	*11*	$\frac{p}{p+0.5q}$	$\frac{0.5q}{p+0.5q}$	0	0.5q/(p+0.5q)
11	*11*	*12*	0	1	0	1
11	*12*	*11*	$\frac{p}{p+0.5q}$	$\frac{0.5q}{p+0.5q}$	0	0.5q/(p+0.5q)
11	*12*	*12*	p	q	0	q
11	*12*	*22*	0	1	0	1
11	*22*	*12*	$\frac{p}{p+0.5q}$	$\frac{0.5q}{p+0.5q}$	0	0.5q/(p+0.5q)
11	*22*	*22*	0	1	0	1
12	*11*	*11*	$\frac{p}{p+0.5}$	$\frac{0.5}{p+0.5}$	0	q *
12	*11*	*12*	0	$\frac{0.5}{q+0.5}$	$\frac{q}{q+0.5}$	p+2q **
12	*12*	*11*	$\frac{p}{p+0.5}$	$\frac{0.5}{p+0.5}$	0	q *
12	*12*	*12*	0.5p	0.5	0.5q	0.5+q
12	*12*	*22*	0	$\frac{0.5}{q+0.5}$	$\frac{q}{q+0.5}$	p+2q **
12	*22*	*12*	$\frac{p}{p+0.5}$	$\frac{0.5}{p+0.5}$	0	q *
12	*22*	*22*	0	$\frac{0.5}{q+0.5}$	$\frac{q}{q+0.5}$	p+2q **

22	*11*	*11*	0	1	0	1
22	*11*	*12*	0	$\frac{0.5p}{q+0.5p}$	$\frac{q}{q+0.5p}$	(0.5p+2q)/(0.5p+q)
22	*12*	*11*	0	1	0	1
22	*12*	*12*	0	p	q	p+2q
22	*12*	*22*	0	$\frac{0.5p}{q+0.5p}$	$\frac{q}{q+0.5p}$	(0.5p+2q)/(0.5p+q)
22	*22*	*12*	0	1	0	1
22	*22*	*22*	0	$\frac{0.5p}{q+0.5p}$	$\frac{q}{q+0.5p}$	(0.5p+2q)/(0.5p+q)

For * we use the equivalence P(11)=p and P(12)=q for the allele dosage formula instead P(11)=p/(p+0.5) and P(12)=0.5/(p+0.5) to make sure that the allele dosage is in the same closed interval [0,1] as the integer genotype resulting from column 4-6. Using P(11)=p/(p+0.5) and P(12)=0.5/(p+0.5) would result in an allele dosage interval of [0.33,1].

For ** we use the equivalence P(12)=p and P(22)=q for the allege dosage formula instead P(12)=0.5/(q+0.5) and P(22)=q/(q+0.5) to make sure that the allele dosage is in the same closed interval [1,2] as the integer genotype resulting from column 4-6. Using P(12)=0.5/(q+0.5) and P(22)=q/(q+0.5) would result in an allele dosage interval of [1,1.67].

Table S3. Correlation between true and genomic estimated breeding values in the validation set obtained when estimating SNP effects with Offspring only (TS) or with an augmented training set including imputed dams (TSA)

h^2	No. of Offspring	LowLD_NoSel		LowLD_Sel		HighLD_NoSel		HighLD_Sel	
		TS	TSA	TS	TSA	TS	TSA	TS	TSA
0.05	2000	0.31	0.38	0.63	0.70	0.54	0.57	0.59	0.66
0.05	4000	0.41	0.49	0.70	0.74	0.61	0.65	0.69	0.75
0.05	8000	0.48	0.57	0.76	0.79	0.70	0.75	0.76	0.80
0.05	16000	0.61	0.68	0.80	0.83	0.77	0.81	0.81	0.83
0.10	2000	0.35	0.48	0.67	0.73	0.59	0.68	0.69	0.72
0.10	4000	0.51	0.60	0.77	0.81	0.71	0.76	0.75	0.79
0.10	8000	0.60	0.69	0.81	0.84	0.77	0.82	0.81	0.83
0.10	16000	0.70	0.76	0.86	0.87	0.83	0.86	0.85	0.87
0.15	2000	0.40	0.53	0.75	0.78	0.69	0.76	0.72	0.76
0.15	4000	0.57	0.62	0.79	0.82	0.75	0.80	0.79	0.82
0.15	8000	0.66	0.73	0.82	0.84	0.81	0.86	0.84	0.86
0.15	16000	0.73	0.79	0.87	0.88	0.86	0.89	0.87	0.89
0.20	2000	0.39	0.51	0.76	0.79	0.70	0.77	0.76	0.80
0.20	4000	0.55	0.66	0.79	0.83	0.75	0.80	0.80	0.84
0.20	8000	0.67	0.75	0.83	0.86	0.83	0.87	0.85	0.87
0.20	16000	0.77	0.82	0.88	0.89	0.88	0.91	0.88	0.90
0.25	2000	0.46	0.63	0.76	0.79	0.76	0.79	0.80	0.81
0.25	4000	0.61	0.71	0.80	0.83	0.79	0.84	0.83	0.85
0.25	8000	0.71	0.78	0.84	0.86	0.85	0.88	0.86	0.88
0.25	16000	0.78	0.83	0.88	0.89	0.90	0.92	0.90	0.91
0.30	2000	0.52	0.62	0.77	0.80	0.77	0.82	0.80	0.83
0.30	4000	0.61	0.71	0.82	0.84	0.82	0.86	0.84	0.86
0.30	8000	0.74	0.80	0.85	0.87	0.87	0.89	0.87	0.89
0.30	16000	0.79	0.84	0.89	0.90	0.91	0.92	0.90	0.91
0.35	2000	0.52	0.65	0.77	0.80	0.79	0.83	0.80	0.84
0.35	4000	0.64	0.74	0.82	0.84	0.84	0.86	0.85	0.87
0.35	8000	0.73	0.79	0.85	0.87	0.88	0.90	0.88	0.90
0.35	16000	0.81	0.84	0.89	0.90	0.92	0.93	0.91	0.92
0.40	2000	0.51	0.65	0.78	0.81	0.80	0.84	0.82	0.85
0.40	4000	0.66	0.74	0.83	0.84	0.85	0.88	0.86	0.88
0.40	8000	0.75	0.81	0.86	0.87	0.89	0.91	0.89	0.90
0.40	16000	0.81	0.85	0.89	0.90	0.92	0.94	0.92	0.92
0.45	2000	0.56	0.66	0.78	0.81	0.83	0.86	0.82	0.85
0.45	4000	0.65	0.74	0.83	0.84	0.85	0.89	0.86	0.88
0.45	8000	0.76	0.81	0.86	0.87	0.89	0.91	0.90	0.91
0.45	16000	0.81	0.85	0.89	0.90	0.93	0.94	0.92	0.93
0.50	2000	0.55	0.68	0.78	0.81	0.83	0.86	0.84	0.86
0.50	4000	0.66	0.74	0.83	0.84	0.87	0.89	0.88	0.89
0.50	8000	0.76	0.81	0.86	0.87	0.90	0.92	0.90	0.91
0.50	16000	0.82	0.85	0.90	0.90	0.94	0.94	0.93	0.93
