# Supplementary material for: Enlarging a training set for genomic selection by imputation of un-genotyped animals in populations of varying genetic architecture
Source: Genet Sel Evol. 2013 Apr 26;45(1):12. doi: 10.1186/1297-9686-45-12 (PMC3652763; doi:10.1186/1297-9686-45-12)
Supplement: Additional file 2: Figure S1 — Pair-wise values of r2 against inter-marker distance for all replicates of the four scenarios. Figure S2. Histograms of the frequencies of allele 2 for all replicates of the four scenarios. Figure S3. Distributions of the number of unambiguously imputed loci per Dam for all replicates of the four scenarios. Figure S4. Description: Regression analyses of the percentage increase in accuracy obtained with TSA against the accuracy already obtained with TS across all h2 and numbers of offspring for the four scenarios. [file 1297-9686-45-12-S2.pdf]

Figure S1. Pair-wise values of r2 against inter-marker distance for all replicates of the four scenarios.

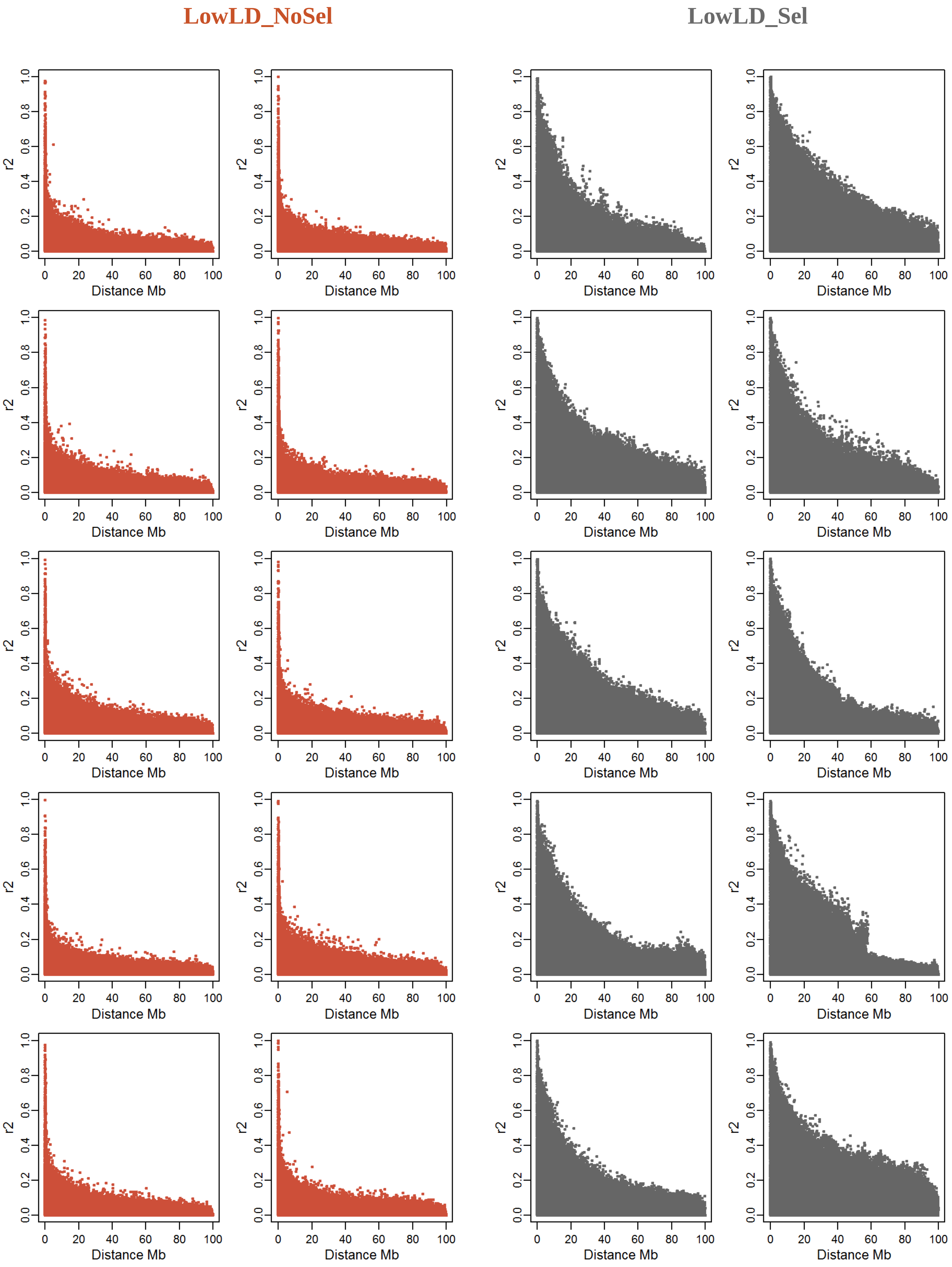
LowLD_NoSel
LowLD_Sel
r2
Distance Mb
0.0
0.2
0.4
0.6
0.8
1.0
0
20
40
60
80
100

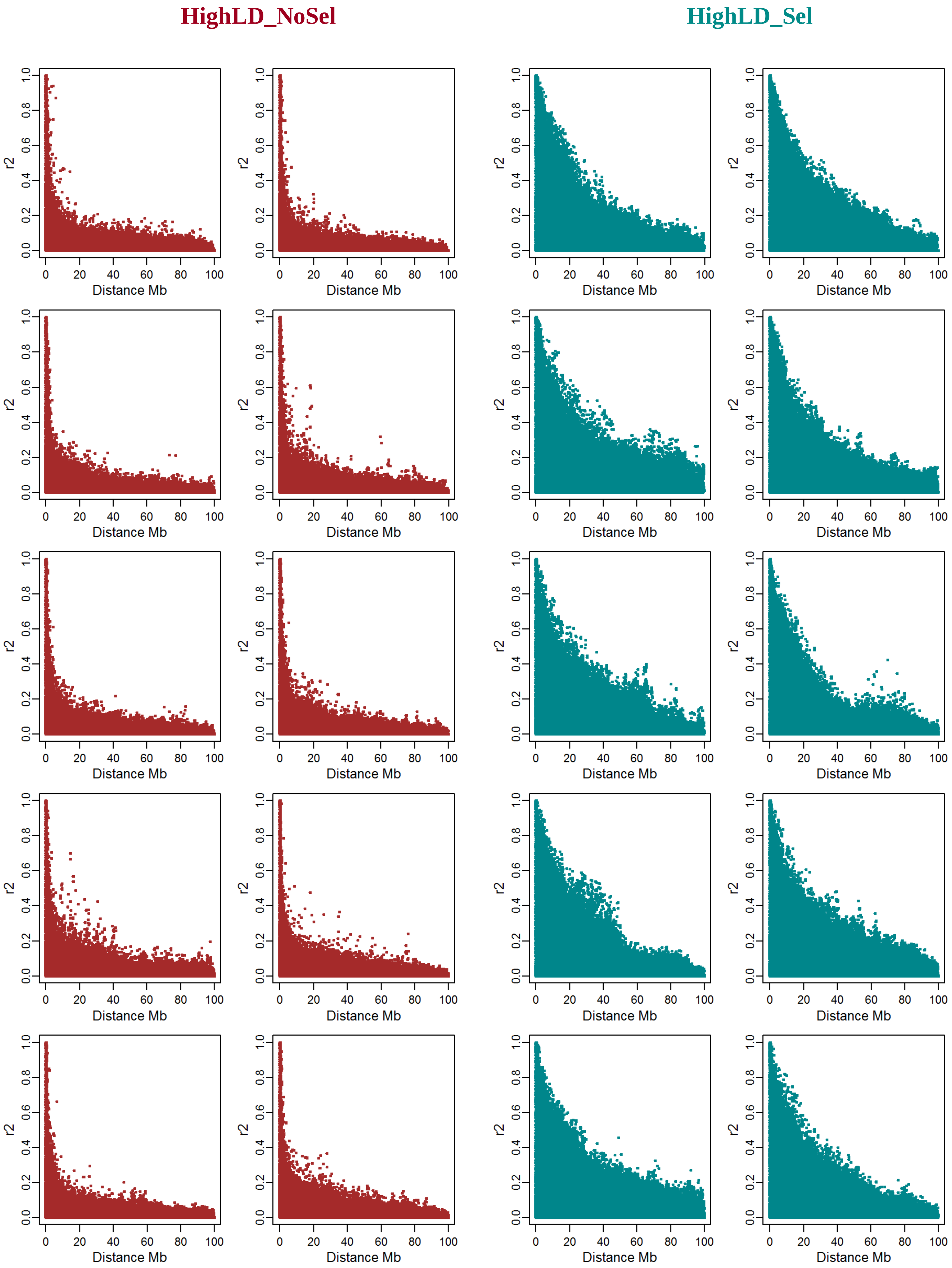
HighLD_NoSel
HighLD_Sel
r2
Distance Mb
0.0 0.2 0.4 0.6 0.8 1.0
0 20 40 60 80 100

Figure S2. Histograms of the frequencies of allele 2 for all replicates of the four scenarios.

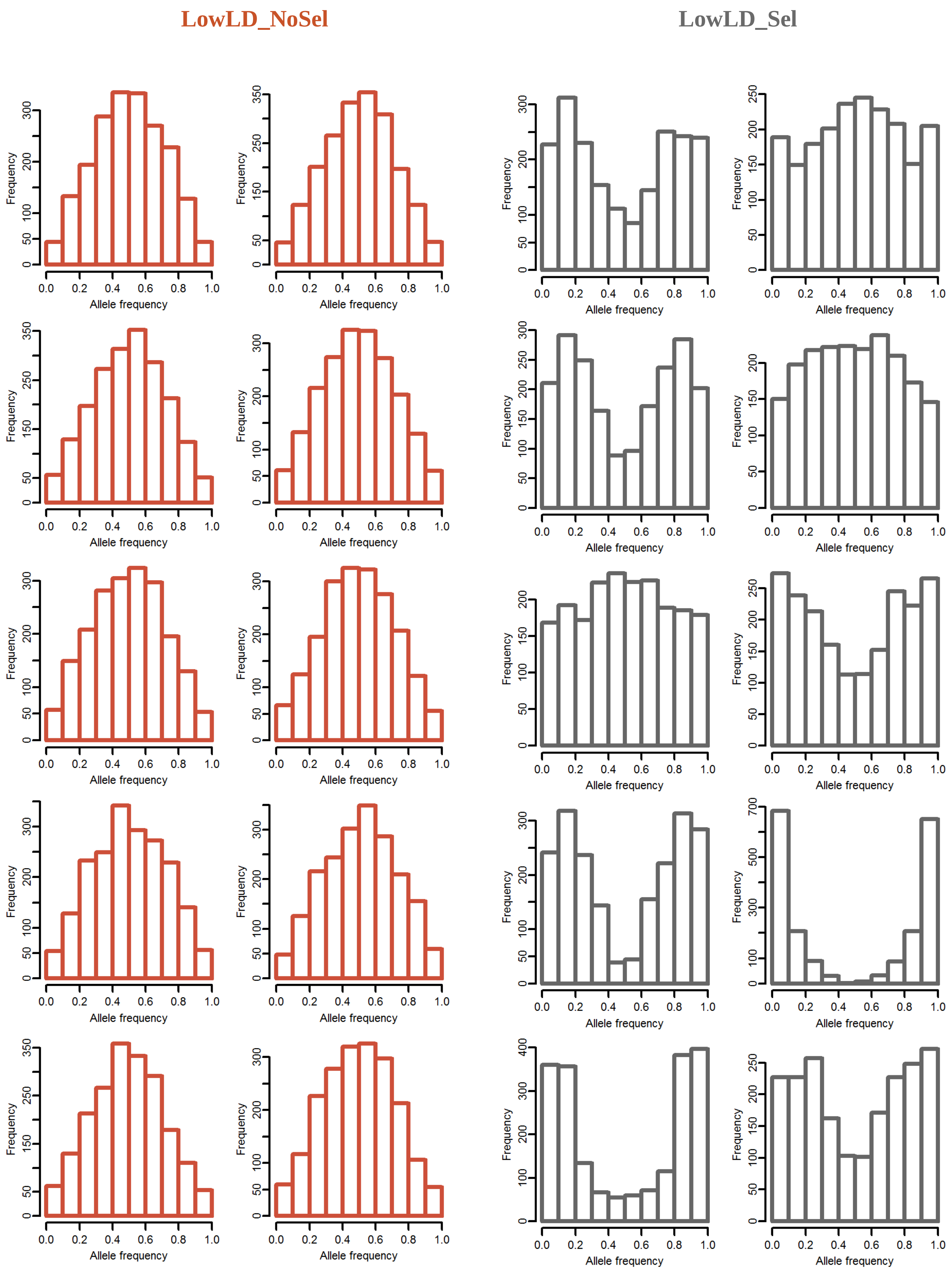
LowLD_NoSel
LowLD_Sel
Frequency
Allele frequency

HighLD_NoSel

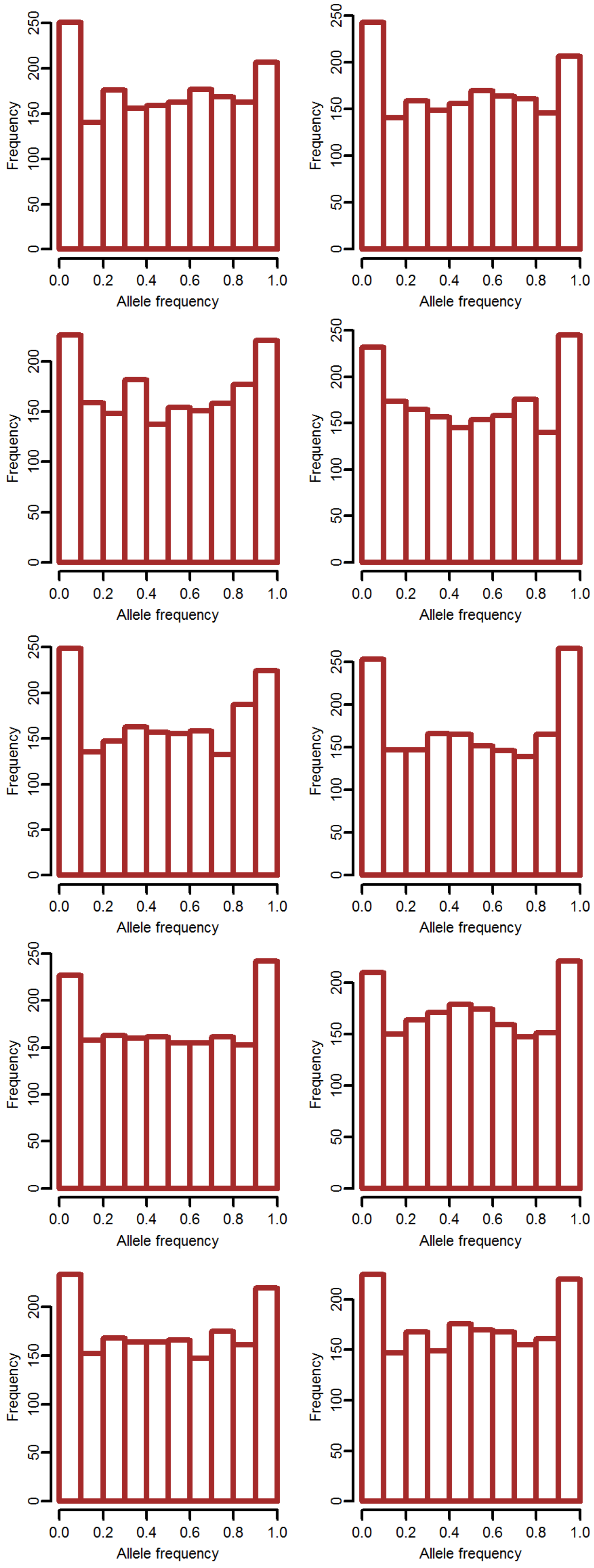

HighLD_Sel

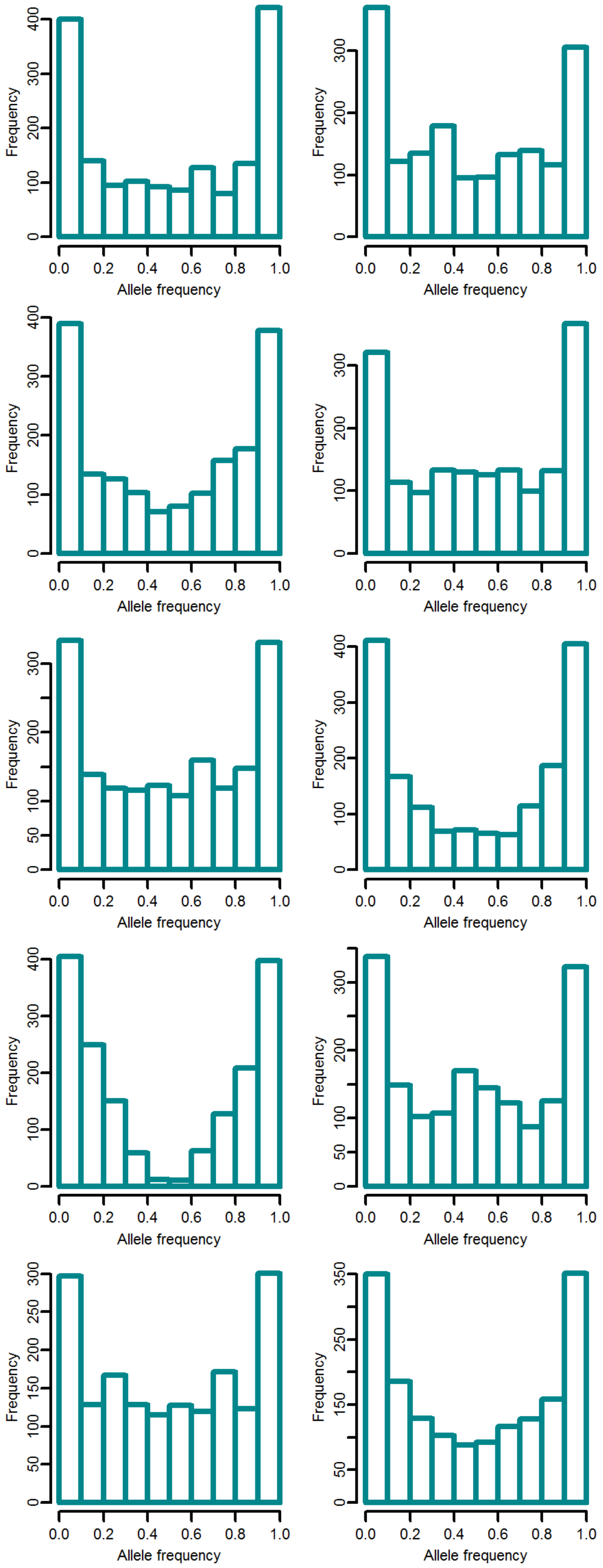

Figure S3. Distributions of the number of unambiguously imputed loci per Dam for all replicates of the four scenarios.

LowLD_NoSel LowLD_Sel

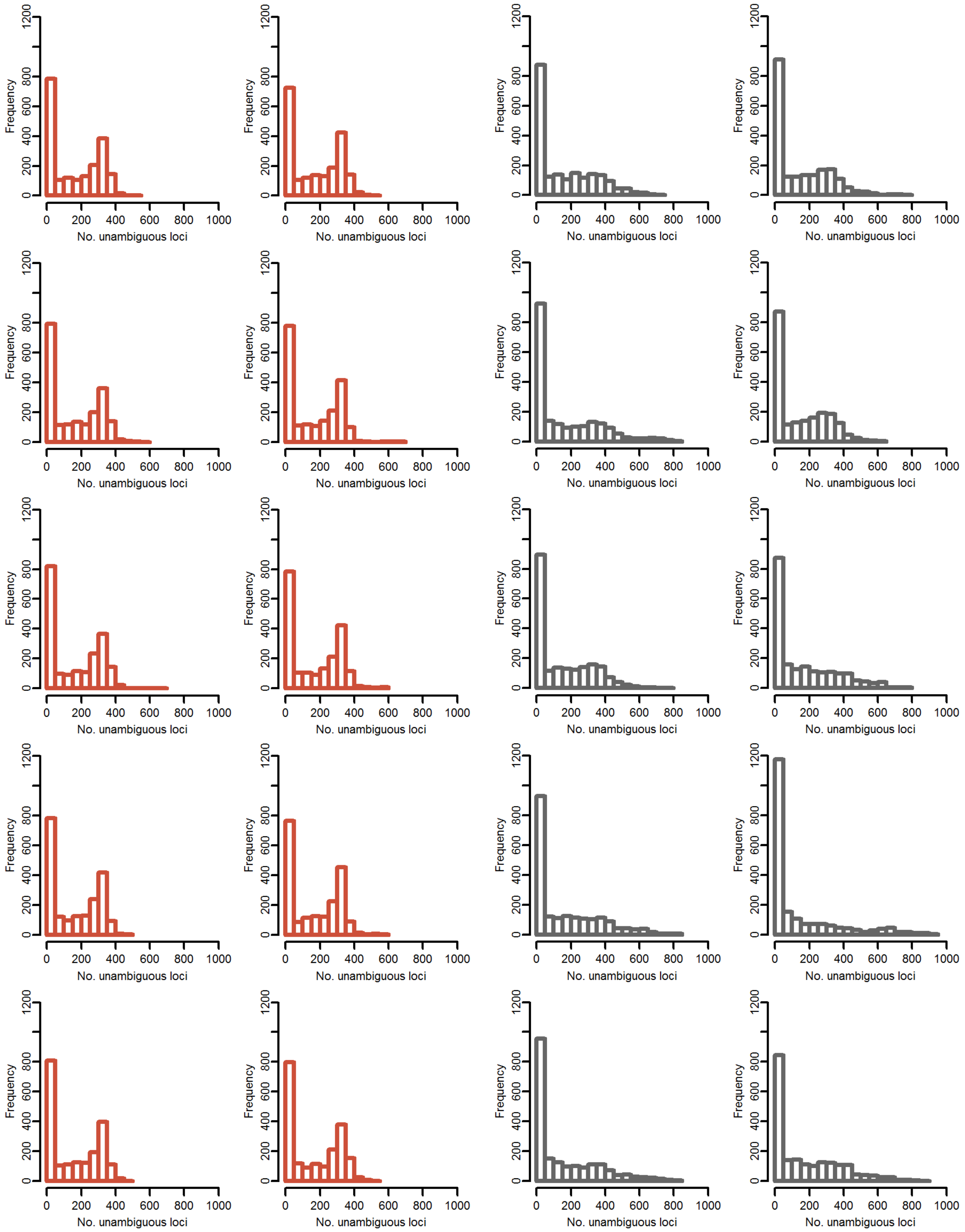

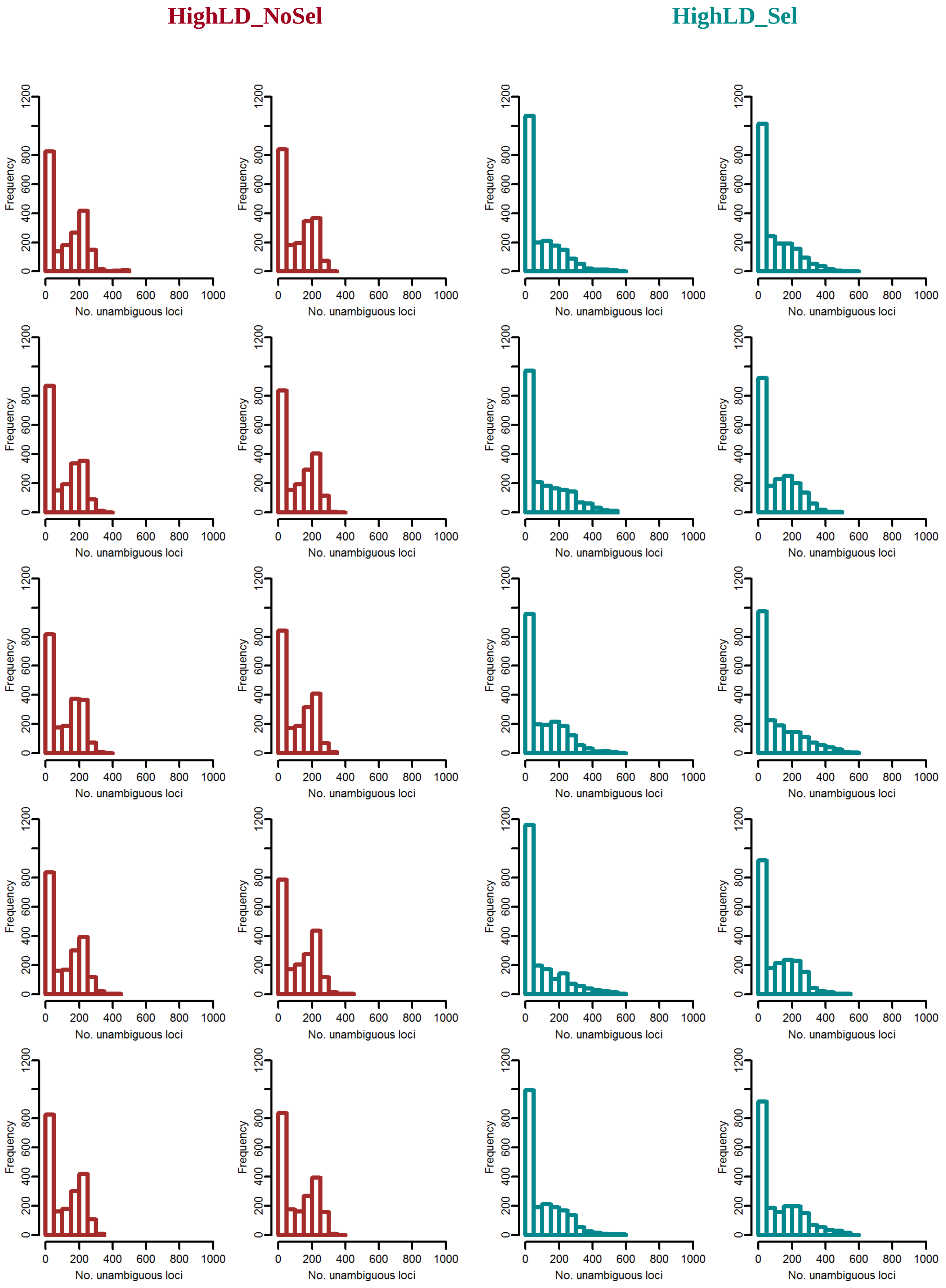
HighLD_NoSel
HighLD_Sel
Frequency
No. unambiguous loci
0
200
400
600
800
1000
1200

Figure S4. Regression analyses of the percentage increase in accuracy obtained with TSA against the accuracy already obtained with TS across all h2 and numbers of offspring for the four scenarios.

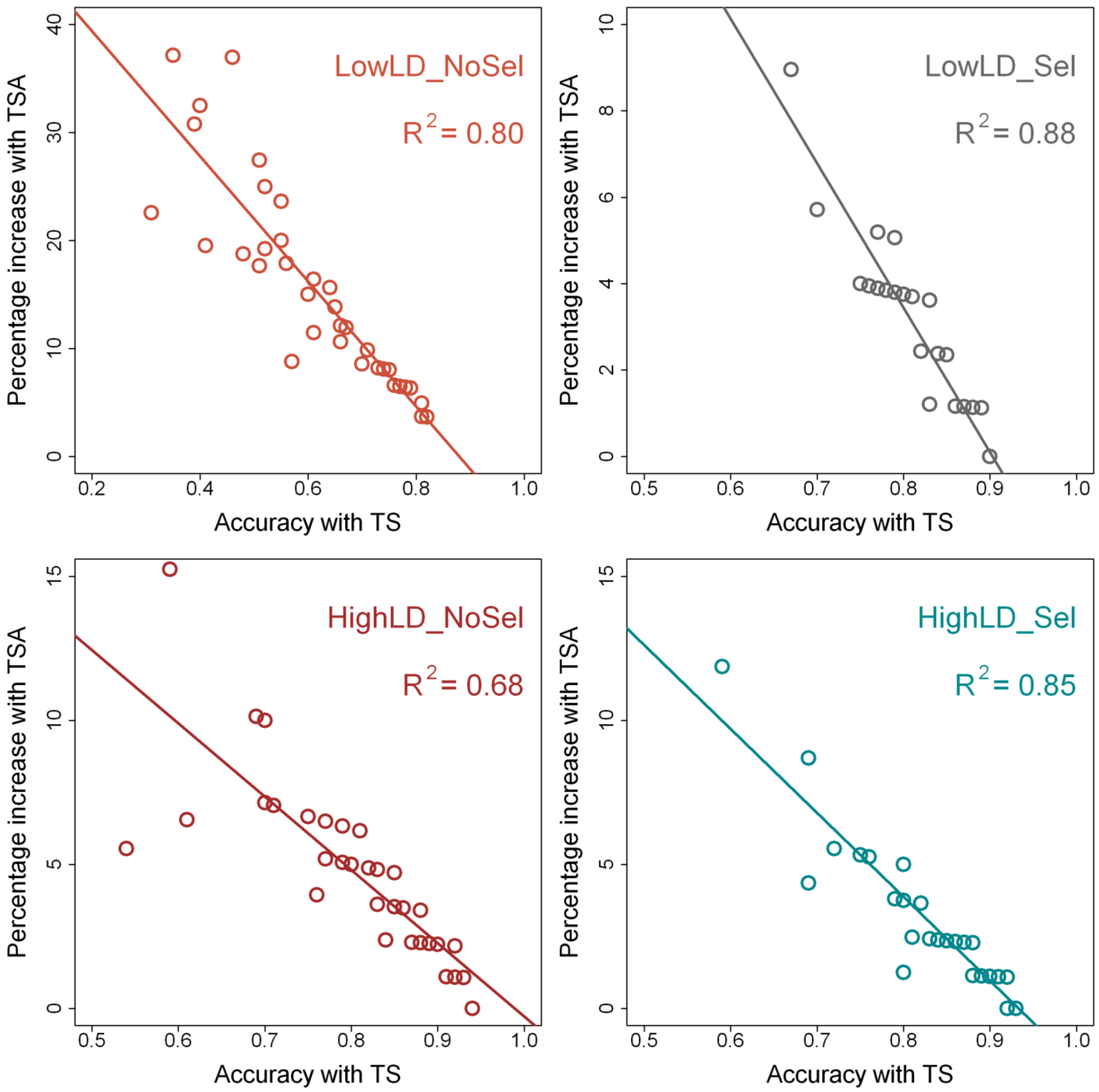

LowLD_NoSel
R2= 0.80
LowLD_Sel
R2= 0.88
HighLD_NoSel
R2= 0.68
HighLD_Sel
R2= 0.85
Percentage increase with TSA
Accuracy with TS
